# The Positive Impact of a Novel Air Purification Technology on Environmental and Clinical Outcomes

**DOI:** 10.1093/geroni/igab046.535

**Published:** 2021-12-17

**Authors:** Susan Schlener, Kathryn Worrilow

**Affiliations:** 1 Phoebe Ministries, Allentown, Pennsylvania, United States; 2 LifeAire Systems, Allentown, Pennsylvania, United States

## Abstract

Reducing sources of illness improves resident care. An advanced air purification technology (AAPT) was designed to destroy the DNA and RNA of all bacteria, fungi and viruses, rendering them non-infectious and to remediate volatile organic compounds (VOCs). This study compares the biological, fungal and VOC loading using the AAPT to standard high efficiency particulate air (HEPA) filtration. It was hypothesized that the AAPT would be associated with reductions in airborne and surface pathogens, VOCs and improved clinical metrics. A control floor with HEPA filtration and study floor with AAPT remediation were studied. Measurements of total VOCs and airborne and surface bacteria and fungi were measured in five locations on each floor. The facility acquired infection (FAI) rate, the number of infections divided by total patient days, showed a 57% difference between the control floor (2.33 FAIs/month) and the study floor (1.00 FAIs/month) and a decrease of 39.75% pre-installation (1.66 FAIs/month) to post-installation (1.00 FAIs/month). The viable pathogen loading measured on the study floor was reduced from an average of 483.8 colony forming units (CFU)/m3 pre-installation to an average of 56 CFU/m3 post-installation. VOCs were reduced from an average of 641.66 parts per billion (PPB) to 64.96 PPB and viable surface bacteria from an average of 110.6 CFU/m3 to 97.2 CFU/m3. The AAPT significantly reduced levels of infectious airborne and surface pathogens and VOC levels. As a result, residents on the AAPT floor demonstrated significant improvements in FAI rates. The findings support the hypothesis that environmental factors impact resident wellness.

